# Correlation of microbiota in the gut of fish species and water

**DOI:** 10.1007/s13205-020-02461-5

**Published:** 2020-10-12

**Authors:** Ao Zeng, Kai Tan, Ping Gong, Ping Lei, Zhaohui Guo, Shengping Wang, Shufeng Gao, Yinghua Zhou, Yan Shu, Xiaoling Zhou, Dong Miao, Fajiao Zeng, Huizhi Liu

**Affiliations:** 1 Department of Animal Nutrition, Hunan Institute of Microbiology, No 18 Xinkaipu Road, Changsha, 410009 Hunan China; 2 College of Fisheries and Life Science, Shanghai Ocean University, Shanghai, 201306 China

**Keywords:** Fish species, Gut microbiota, 16S rRNA, Loudi

## Abstract

To analyze the intestinal microbiota diversity of several important economic fishes in the Loudi area and its correlation with the microbiota of water environment, the high-throughput sequencing based on the bacteria 16S rRNA was used to analyze the intestinal microbiota diversity in fish intestines and water. The results revealed that half of the OTUs in the water sample could be detected in the fish intestine, the proportion of shared OTUs in the intestines of *Hypophthalmichthys molitrix* and water samples was only 22%, and the unique OTU in the LC group was relatively the highest in the fish intestinal group. It can be seen from the analysis in NMDS analysis, the distance between *Hypophthalmichthys molitrix* group and water group is relatively farthest. *Ctenopharyngodon idellus* has the highest microbiota richness and diversity (*P* < 0.05), while the water samples have the lowest microbiota richness (*P* < 0.05). Firmicutes, *Methylocaldum* and *Bacillus* are the prevalent taxonomic unit in the *Aristichthys nobilis* and *Carassius auratus* groups, *Anaerospora* is the prevalent genera in the *Hypophthalmichthys molitrix* group, Proteobacteria and Cyanobacteria have a high relative abundance ratio in the *Ctenopharyngodon idellus* group, and the prevalent taxonomic unit in the water sample group are *Phenylobacterium*, Bacteroidetes and Actinobacteria. In conclusion, fish species have different prevalent microbiota. There are a strong correlation between fish intestinal microbiota and the water environment, and the fish with a weak correlation is *Hypophthalmichthys molitrix*. Results of this study will contribute to the prevention and treatment of fish diseases and the fish ecological culturing in Loudi area.

## Introduction

The intestinal microbiota of fish are much more simpler than mammals (Mitra et al. 2014; Okadinya et al. 2013). Similarly, due to the limitations of fish physiological conditions, in which the evolution of the intestine of fish is not complete, the digestive enzymes secreted by intestinal microorganisms and the regulating functions of intestinal microorganisms on fish immunity are particularly important. Intestinal physiological microbiota, fish body and the surrounding water environment constitute a ecosystem, which is interdependent and mutually restricted. The balance of this system is the key factor to maintain the health of fish.

The gut microbiota of fish is affected by a variety of factors. Among these, the main factors are the type of fish and the waters it inhabits, while other factors include the bait, development stage, water temperature and physiological conditions (Jiang et al. 2016). At present, there have been many reports on the microbiota composition of freshwater fish. Different from the mammalian intestinal microbiota, which are mainly dominated by *bifidobacteria* and *lactobacillus*, the prevalent intestinal microbiota of fish has exhibited huge differences, and even the intestinal prevalent microbiota of the same fish differs in different regions (Nilsson et al. 2016; Chen et al. 2015). Therefore, the study of fish intestinal microorganisms should be systematic or qualitatively researched in fixed culturing conditions.

Pond intensive aquaculture has a short cycle and high yield, which is the main mode of aquaculture in China. However, this model has a high culturing density, and a large amount of bait and medicine are used, which has a great negative impact on disease outbreaks and the surrounding environment (Li et al. 2010). At this stage, the microecosystem comprise of the fish gut, fish body and the surrounding water environment in the Loudi area, which is already very fragile. Therefore, it is necessary to study the gut microbiota of fish in different environmental conditions. The present study aims to analyze the intestinal bacterial diversity of several important economic fishes in the Loudi area and their correlation with the bacterial structure of the water environment, to provide basic data for the prevention and treatment of fish diseases, the use of microbial additives, and the fish ecological culturing in this area.

## Materials and methods

### Collection of samples

The four kinds of experimental fishes included *Hypophthalmichthys molitrix*, *Aristichthys nobilis*, *Ctenopharyngodon idellus* and *Carassius auratus*. The water samples were collected from the same pond at the aquaculture base of Loudi Institute of Fishery Science. The pond has a water depth of 2.0 m and an area of 1.5 hm^2^. Commercial fish feed (Grass Carp 707 series, Haida Group) was fed for two times a day (08:00 and 15:00 h). The fish and water samples were taken at 07:00 h on June 25, 2019. The water temperature was 27.8 °C, pH was 6.95, and the dissolved oxygen was 3.35 mg L^−1^. In the tira net catch, three healthy fishes of the same size were randomly selected from each type for weighing, which included *Ctenopharyngodon idellus* 0.60 kg, *Carassius auratus* 0.21 kg, *Hypophthalmichthys molitrix* 0.35 kg, and *Aristichthys nobilis* 0.31 kg. At the same time, water samples were collected at 1.0 m below the water surface from three randomly selected points in the fish pond. Then, these samples were immediately ice-bathed and shipped back to the laboratory.

### Extraction of contents and collection of bacteria in water samples

After the fish samples were returned to the laboratory, fish samples were anesthetized with eugenol (1:10,000), and the surface of the fish was successively rinsed with sterile water and 70% ethanol. Then, the contents of the foregut section of all samples were aseptically collected in a sterile operation box. Twelve intestinal contents were sampled as *Hypophthalmichthys molitrix* (LC1–LC3), *Aristichthys nobilis* (YC1–YC3), *Ctenopharyngodon idellus* (CC1–CC2), and *Carassius auratus* (JC1–JC3).

After the water sample was transported back to the laboratory, the water sample was filtered with a sterile filter(0.2 μm) under a sterile environment. Each sample was filtered by 2 L, and the filter membrane was stored for future use. The sample numbers were SS, SZ and SX.

### Extraction of DNA

The water environmental DNA and content DNA were extracted using a Tiangen stool DNA extraction kit (TIANamp Stool DNA Kit). The specific operations were based on the kit instructions. The extracted DNA was analyzed by agarose gel electrophoresis, and the concentration and purity of the total DNA were detected using an ultra-micro ultraviolet spectrophotometer. Finally, the DNA was stored at − 20 °C until use.

### PCR amplification and preparation of the sequencing library

The total sample DNA was used as the template for the PCR amplification of the bacterial 16S rRNA gene in the V3 + V4 region. The V3 + V4 variable region common primer was synthesized by Shanghai Personal Biotechnology Co., Ltd. The primer sequence was 338F: 5′-ACTCCTACGGGAGGCAGCA-3′ and 806R: 5′-GGACTACHVGGGTWTCTAAT-3′. PCR amplification system: 25 μL: 5 × reaction buffer 5 μL, 5 × GC buffer 5 μL, dNTP (2.5 mM) 2 μL, forward primer (10 μM) 1 μL, reverse primer (10 μM) 1 μL, DNA template 2 μL, ddH_2_O 8.75 μL, and Q5 DNA polymerase 0.25 μL. Amplification parameters: initial denaturation at 98 °C for two minutes, denaturation at 98 °C for 15 s, annealing at 55 °C for 30 s, extension at 72 °C for 30 s, and final extension at 72 °C for 5 min; 29 cycles. The PCR products were detected by agarose gel electrophoresis and purified using the agarose gel recovery kit (Shao et al. 2020). The fluorescence reagent used was the quant-it PicoGreen dsDNA Assay Kit, and the quantitative instrument performed using a microplate reader (BioTek, FLx800).

The sequencing library was prepared according to the instructions of the TruSeq Nano DNA LT Library Prep Kit (Illumina). The library concentration was > 2 nM. The amplified V3 + V4 variable region of the 16S rRNA was subsequently sequenced using the Illumina MiSeq platform (Frasergen Co., Ltd.).

### Bioinformatics analysis

The quality control processing, such as double-end deduplication, splicing and chimera removal, was performed on the raw data after the disembarkation, to obtain high-quality valid tags. Then, the sequences obtained above were merged and clustered into operational taxonomic units (OTUs) with 97% sequence similarity. The sequence with the highest abundance in each classification unit was selected as the representative sequence of the unit (Edgar 2010). Finally, the sample OTUs were analyzed for abundance, α-diversity, β-diversity, and the bacterial community structure at each classification level.

## Results

### High-throughput data statistics

**Fig. 1:**
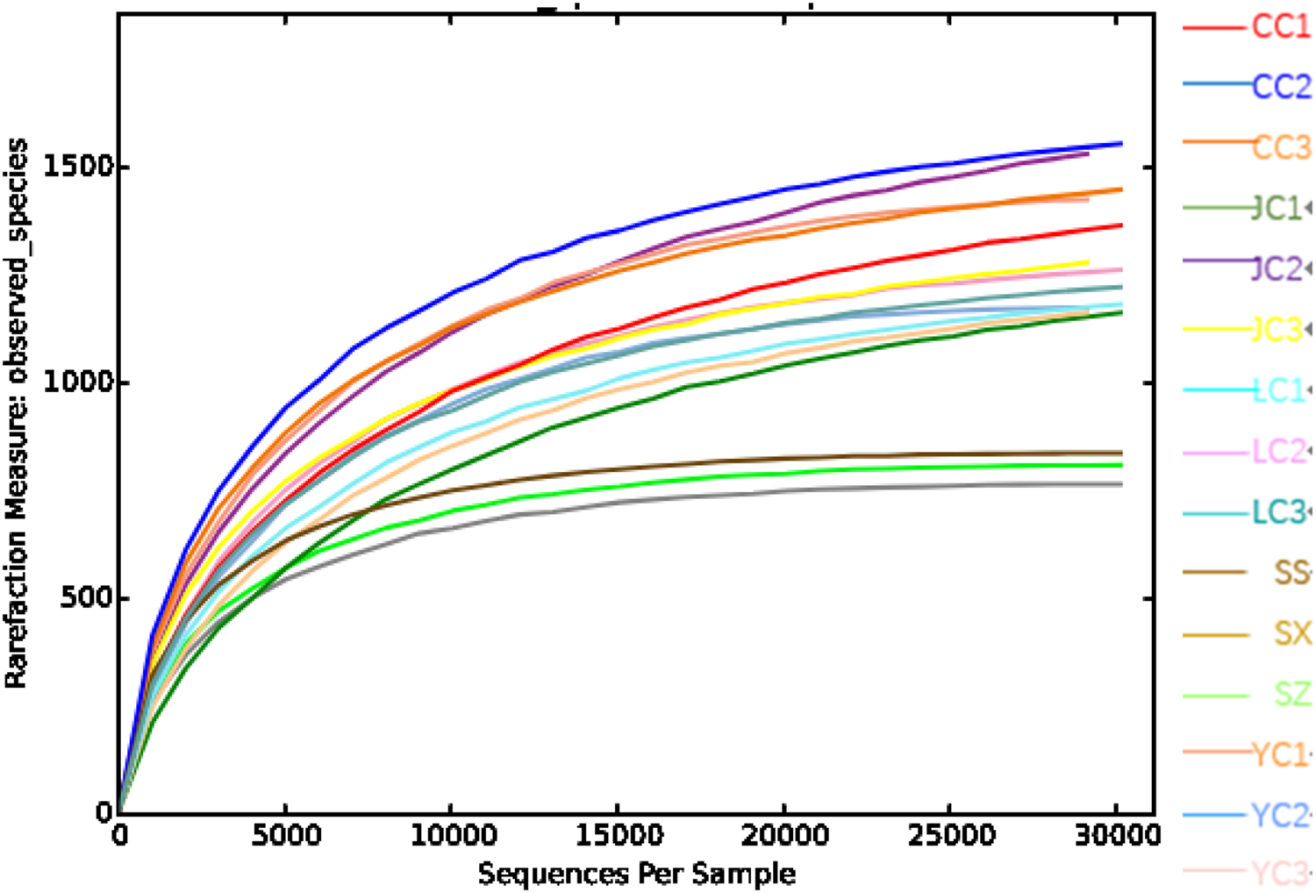
Obeserved species sparse graph. The abscissa represents the sequences randomly selected per sample, and the ordinate represents the number of OTU found at corresponding depth. As the sequencing quantity increased, the number of species found in each sample tended to be flat. *Hypophthalmichthys molitrix* group (LC1–LC3), *Aristichthys nobilis* group (YC1–YC3), *Ctenopharyngodon idellus* group (CC1–CC2), and *Carassius auratus* group (JC1–JC3), Water group (SS, SZ, SX)

For the present study, the Illumina MiSeq platform was used for the paired-end sequencing of DNA fragments. After the initial screening of the original sequencing data, the chimera and question sequences were removed, and the sequence quantity for the subsequent analysis of each sample was obtained. As shown in Table 1, the valid tags of each sample were greater than 40,000. Based on the dilution curve obtained by randomly selecting a certain number of sequencing sequences and the corresponding number of species (Fig. 1), it can be observed from Table 1 and Fig. 1 that as the sequencing quantity increased, the number of species found tended to be flat, indicating that the sequencing depth is enough to reflect the diversity contained in the present samples.

**Table 1:** Statistical table for the sequencing volume

Sample	Sequencing volume
JC1	66,818
JC2	54,659
JC3	59,871
CC1	57,612
CC2	59,733
CC3	56,849
YC1	52,718
YC2	44,459
YC3	56,536
LC1	54,347
LC2	59,426
LC3	59,726
SS	62,010
SZ	57,476
SX	53,575

### Shared OTU analysis

The obtained sequences were merged, and the OTUs were divided according to 97% sequence similarity using the UCLUST sequence comparison tool of the QIIME software (Edgar 2010). A total of 4839 OTUs were obtained. The number of OTUs could represent the richness of the species. Among these, the OTUs detected in the JC, CC, YC, LC and S groups were 2416, 2575, 1915, 1548 and 1015, respectively.

**Fig. 2:**
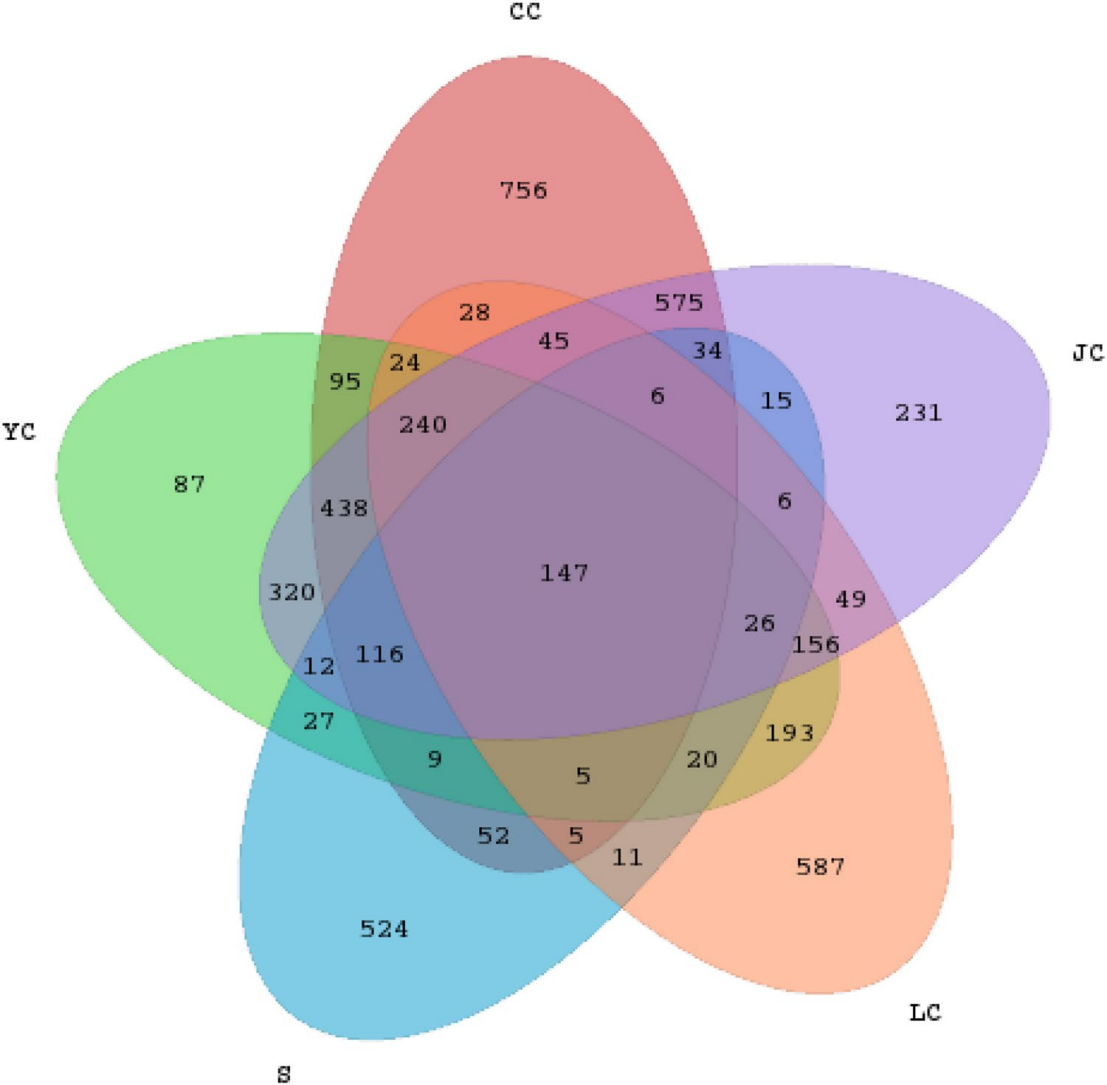
Venn diagram. The number of each block indicates the number of shared or unique OTUs for the group contained in the block. The shared OTU for the CC and JC groups was 1601, the shared OTU for the YC and LC groups was 811, the shared OTU for the CC and YC groups was 1047, the shared OTU for the CC and LC groups was 494, the shared OTU for the CC and S groups was 362, the shared OTU for the S and JC groups was 226, the shared OTU for the S and YC groups was 362, the shared OTU for the YC and JC groups was 1455, and the shared OTU for the LC and JC groups was 528. *LC* *Hypophthalmichthys molitrix* group, *CC* *Ctenopharyngodon idellus* group, *YC* *Aristichthys nobilis* group, *JC* *Carassius auratus* group, *S* water group

The R software was used to calculate the number of shared OTUs in each group based on the obtained OTU abundance matrix, and the proportion of shared and unique OTUs in each group was visually presented by a Venn diagram. Figure 2 shows that the number of shared OTUs for the five groups was merely 147, and the percentage of unique OTUs in the CC, JC, LC, YC and S groups were 29.36, 9.56, 37.92%, 4.54 and 51.63%, respectively, indicating that half of the OTUs in the water sample can be detected from the intestines of inhabited fishes. At the same time, there were some differences in the unique OTU numbers of these fish groups, and the LC group had the highest unique OTU ratio. From the perspective of the shared OTU in all fish groups, there were relatively more shared OTUs among the CC, JC and YC groups, and there were relatively few shared OTUs between these three groups and the LC group. Furthermore, the unique OTU in the LC group was relatively the highest in the fish intestinal group, indicating that there is a relatively significant difference between the bacteria species in the intestinal contents in the LC group and those in other fish intestinal contents.

### The α-diversity analysis

**Fig. 3:**
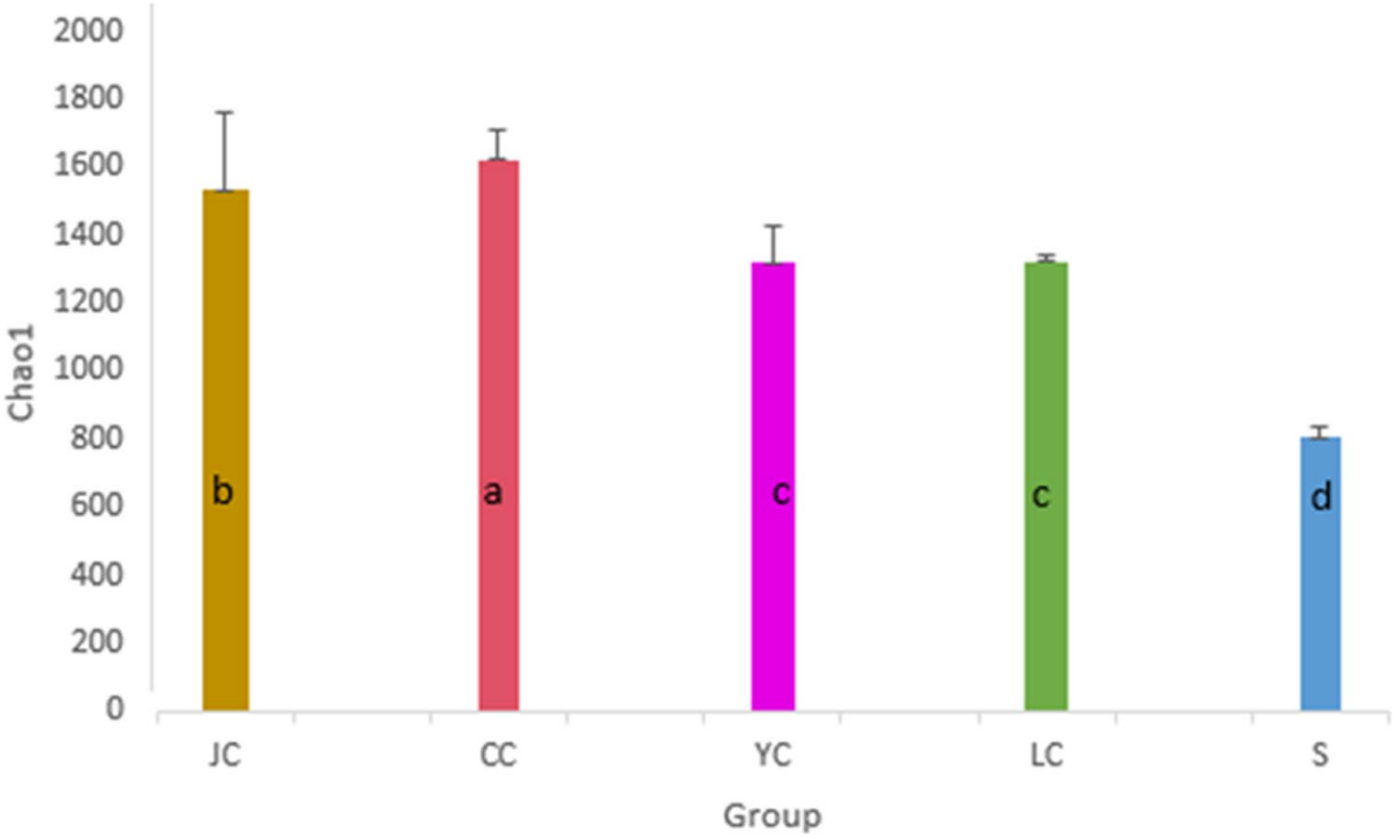
Chao1 index. The Chao1 estimator estimates the number of species that actually exist in the community by counting the number of OTUs detected once and twice in the community. The different lowercase letters indicate the significant difference (*P* < 0.05). *LC* *Hypophthalmichthys molitrix* group, *CC* *Ctenopharyngodon idellus* group, *YC* *Aristichthys nobilis* group, *JC* *Carassius auratus* group, *S* water group

**Fig. 4:**
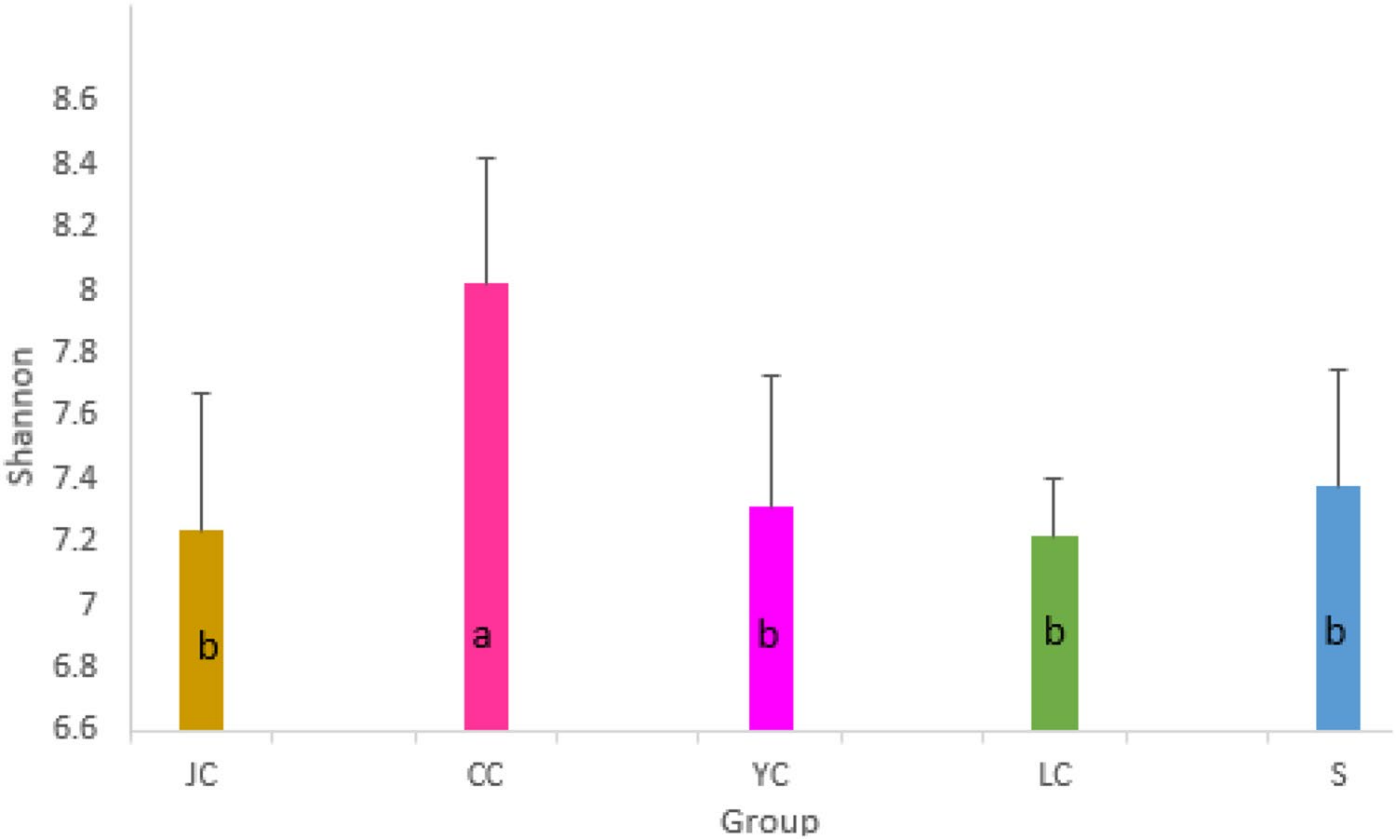
Shannon diversity index. The higher the Shannon index value, the higher the diversity of the community. The different lowercase letters indicate the significant difference (*P* < 0.05). *LC* *Hypophthalmichthys molitrix* group, *CC* *Ctenopharyngodon idellus* group, *YC* *Aristichthys nobilis* group, *JC* *Carassius auratus* group, *S* water group

The Chao1 estimator estimates the number of species that actually exist in the community by counting the number of OTUs detected once and twice in the community (i.e., “Singleton” and “Doubleton”). Unlike Chao1, the Shannon–Wiener index takes into account the richness and evenness of colonies. The higher the Shannon index value, the higher the diversity of the community. It can be observed from the microbiota richness of each group that there was a significant difference (Fig. 3). Group S (water sample group) had the lowest microbiota richness, when compared to the fish group (*P* < 0.01). YC and LC had similar richness (*P* > 0.05), and were lower than JC and CC (*P* < 0.01). The richness in the CC group was higher than that in the JC group (*P* < 0.05). Therefore, CC > JC > YC and LC > S can be considered in terms of microbiota richness. According to the Shannon index in Fig. 4, the diversity of the CC group was significantly higher than that of the other groups (*P* < 0.05), while the diversity of intestinal content samples in the JC, YC and LC groups and environmental samples was not significantly different (*P* > 0.05).

### β-diversity analysis

**Fig. 5:**
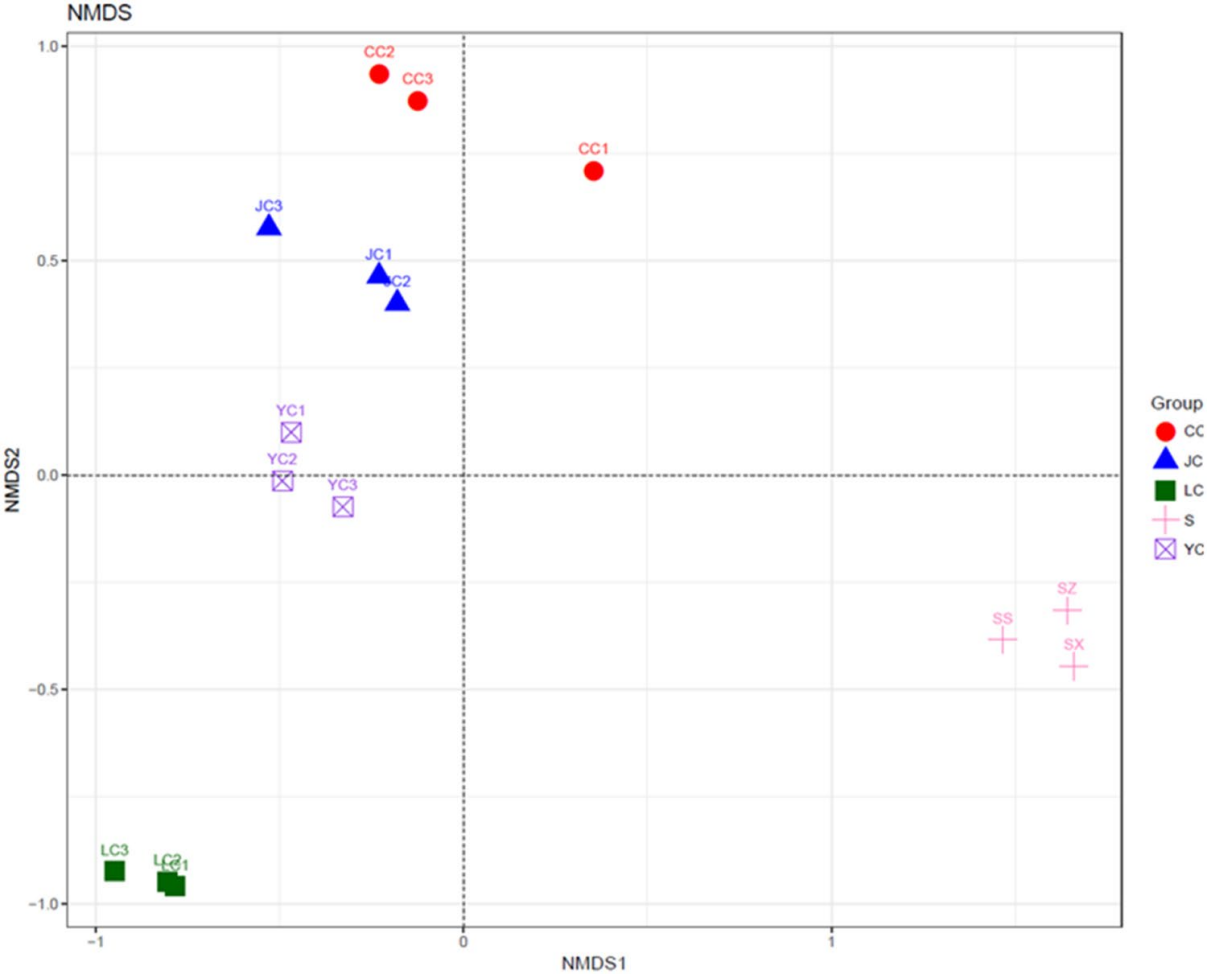
Unweighted UniFrac NMDS analysis. Each point represents a sample, and points of different colors belong to different samples. The closer the distance between the two points, the higher the similarity of the microbial community structure between the two samples, and the smaller the difference. *Hypophthalmichthys molitrix* group (LC1–LC3), *Aristichthys nobilis* group (YC1–YC3), *Ctenopharyngodon idellus* group (CC1–CC2), and *Carassius auratus* group (JC1–JC3), Water group (SS, SZ, SX)

The main purpose of the β-diversity analysis is to determine the similarity of the community structure among different samples (Magali et al. 2013). According to the analysis performed by Unweighted Unifrac NMDS (Fig. 5), the differences within each sample group were small, and these could all gather into a cluster, which can be well-grouped into a ranking chart. The distribution distance between the fish intestinal contents group and water sample group was relatively far, indicating that there are certain differences between the water environment and intestinal content in connection to the bacterial community structure. For the fish intestinal contents group, the YC, JC and CC groups were closer, and these are basically above the horizontal axis, while the three groups were different from the LC group below the horizontal axis.

### Taxonomic composition analysis

**Fig. 6:**
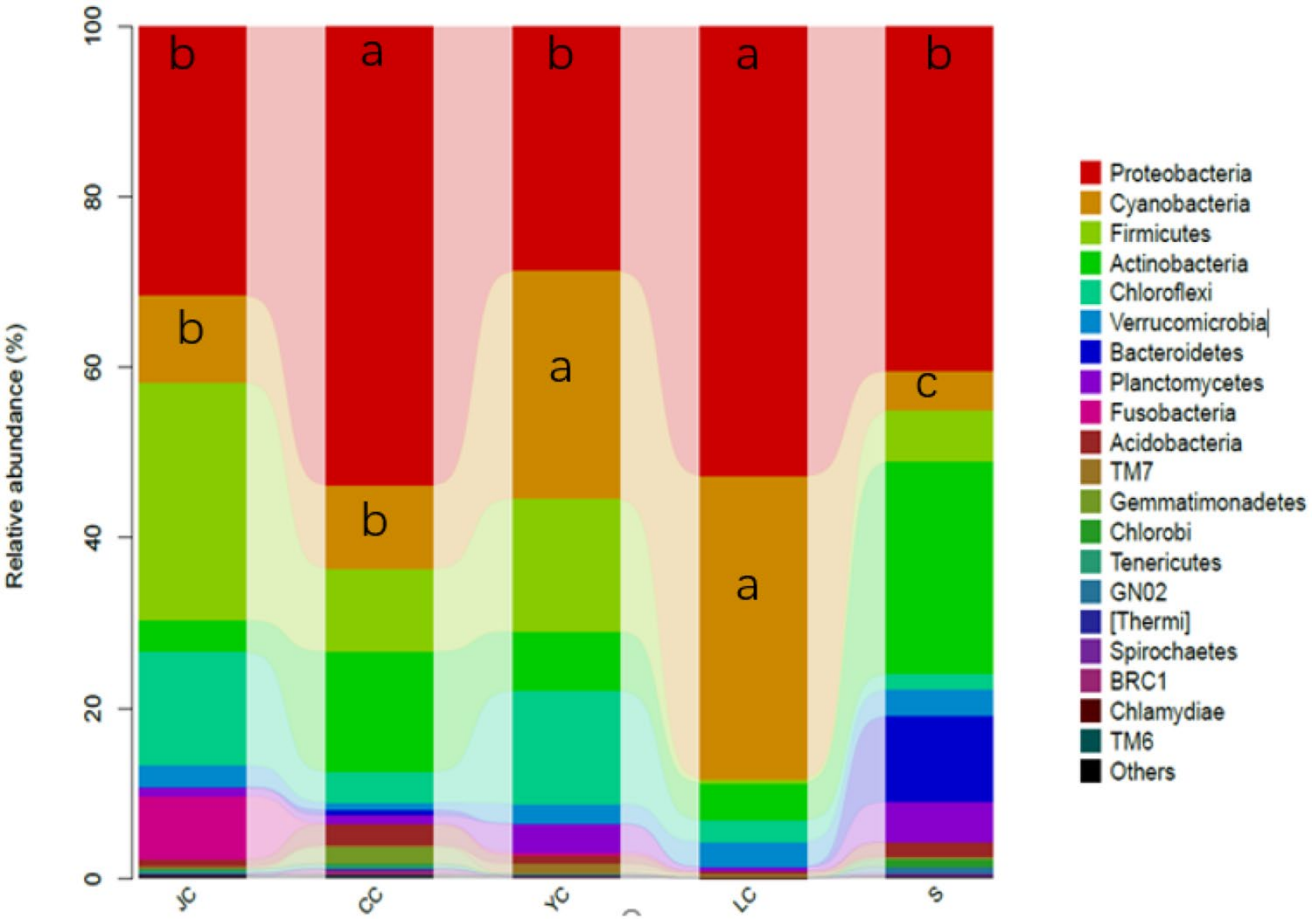
Bacterial distribution based on the phylum taxonomical level. The different lowercase letters indicate the significant difference (*P* < 0.05). *LC* *Hypophthalmichthys molitrix* group, *CC* *Ctenopharyngodon idellus* group, *YC* *Aristichthys nobilis* group, *JC* *Carassius auratus* group, *S* water group

The QIIME software was used to obtain the taxonomic composition and abundance distribution table of each sample at each level. Figure 6 presents the histogram distribution of the microbiota, ranking the top 20 in relative abundance at the phylum level. Figure 6 shows that the fish intestines and water samples mainly included Proteobacteria, Cyanobacteria, Firmicutes, Actinobacteria, Chloroflexi, Verrucomicrobia and Bacteroidetes. Proteobacteria were the highest in abundance in each group, accounting for more than 30%. In comparing the top two relative abundance phylum among groups, the relative abundance ratio of Proteobacteria in the LC and CC groups was significantly higher, when compared to the other groups (*P* < 0.05), and the LC and YC groups had a higher relative abundance ratio of Cyanobacteria (*P* < 0.05).

**Fig. 7:**
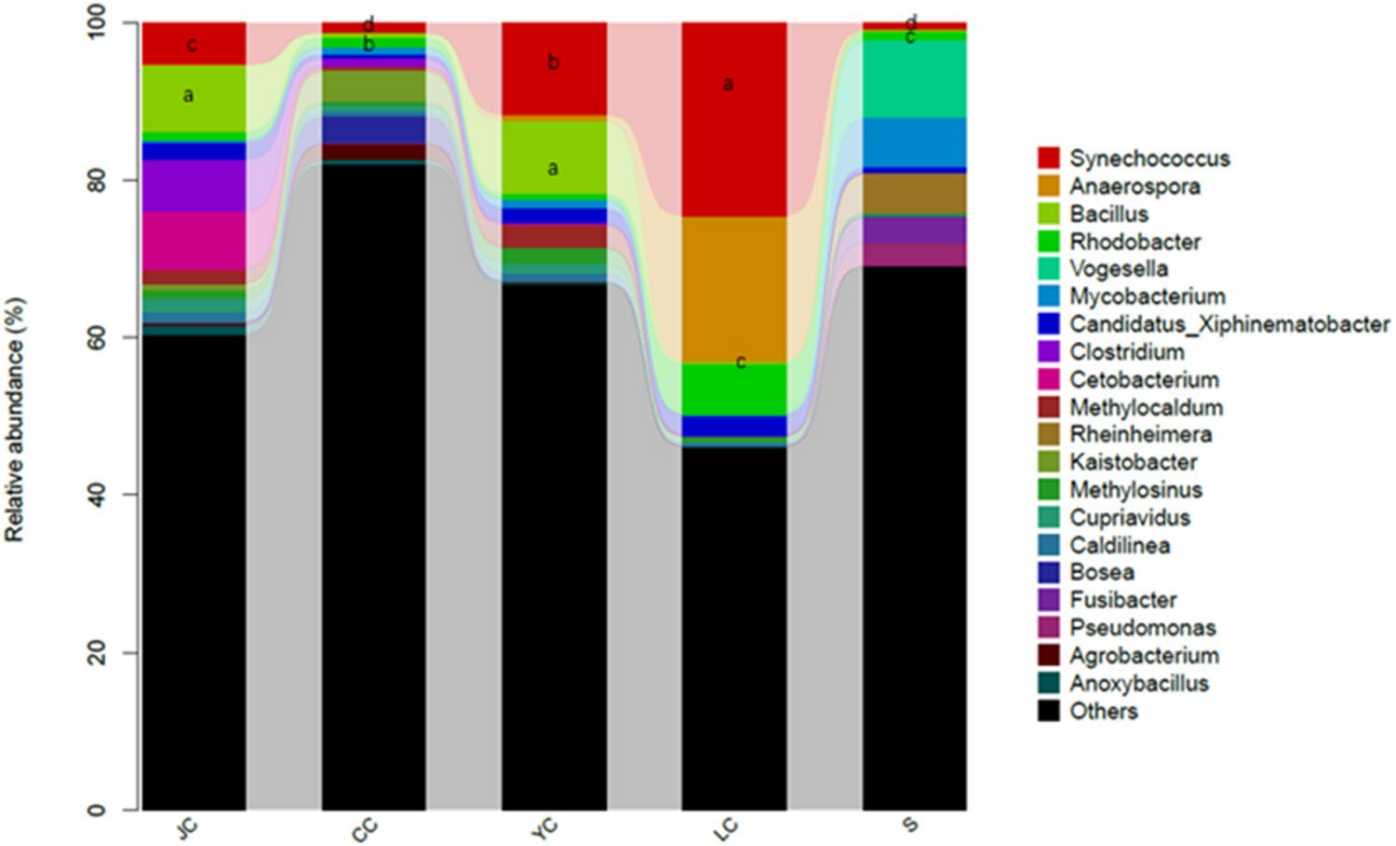
Bacterial distribution based on the genus taxonomical level. The different lowercase letters indicate the significant difference (*P* < 0.05). *CC* *Ctenopharyngodon idellus* group, *YC* *Aristichthys nobilis* group, *JC* *Carassius auratus* group, *S* water group

Figure 7 presents the histogram distribution of microbiota, ranking the top 20 in relative abundance at the genus level. The fish intestines and water samples mainly included *Synechococcus*, *Anaerospora*, Bacillus, *Rhodobacter* and *Vogesella*. In comparing the top two relative abundance genera among groups, *Synechococcus* in the LC group had the highest relative abundance (*P* < 0.05), while *Bacillus* in the YC and JC groups had a relatively high abundance (*P* < 0.05). In the water sample group, the relative abundance of the two genera was the lowest (*P* < 0.05), indicating that the two genera were more suitable for growth in fish intestines.

### Taxonomic composition difference analysis between groups

According to the composition and sequence distribution of each sample at each taxonomic level, the statistical algorithm of Metastats (https://metastats.cbcb.umd.edu/) from the Mothur software was used to determine the absolute abundance difference of the top 10 tax in the genus and phylum level among groups (White et al. 2009). The abundance distribution of the most significant differences among groups is presented in the figure below.

**Fig. 8:**
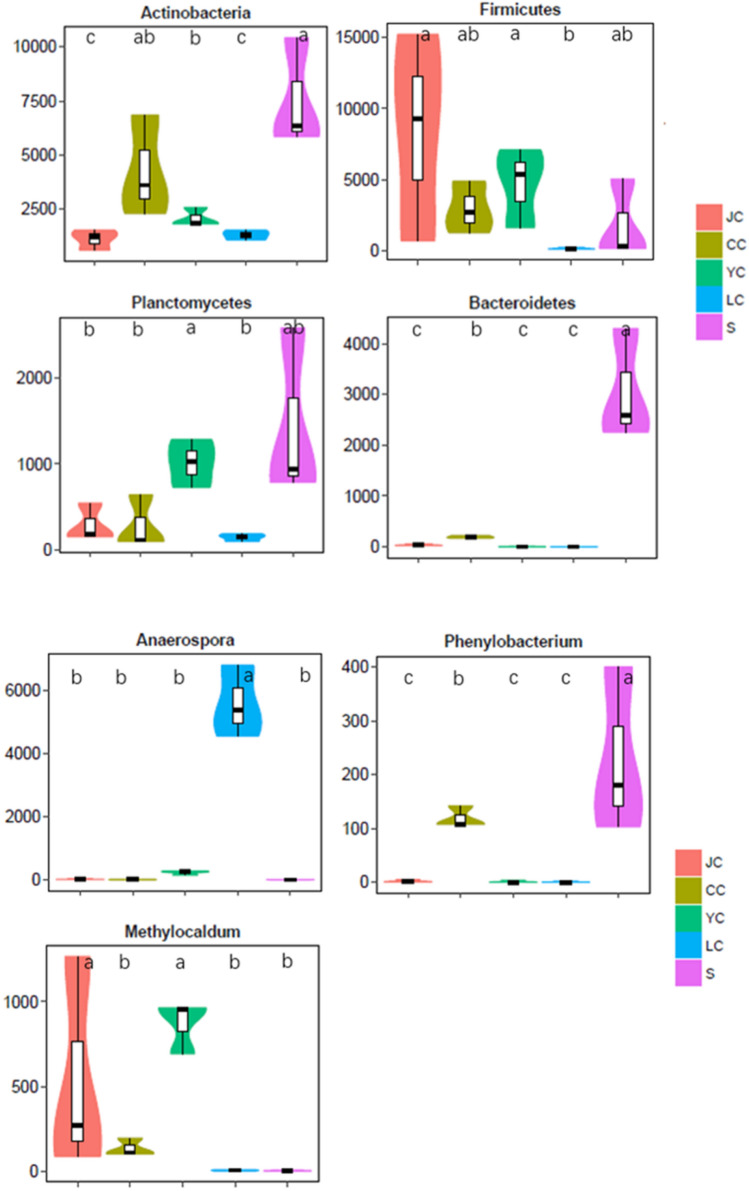
Abundance distribution of taxa with significant differences among groups. The different lowercase letters indicate the significant difference (*P* < 0.05). The abscissa is the taxon with the most significant difference, and the ordinate is the sequence quantity of each taxon in each group. *CC* *Ctenopharyngodon idellus* group, *YC* *Aristichthys nobilis* group, *JC* *Carassius auratus* group, *S* water group

According to Fig. 8, in the fish group, Firmicutes and *Methylocaldum* were the prevalent taxa in the JC and YC groups (*P* < 0.05). *Anaerospora* was the prevalent bacterium in the LC group (*P* < 0.05). The prevalent taxa in the water sample group (*P* < 0.05) were *Phenylobacterium*, Bacteroidetes and Actinobacteria. In conclusion, fish species have different prevalent taxa due to different environments and feeding habits.

## Discussion

*Hypophthalmichthys molitrix*, *Aristichthys nobilis*, *Ctenopharyngodon idellus* and *Carassius auratus* are the most common fish species under the mixed culturing mode in the Loudi area. Among these, *Ctenopharyngodon idellus* is an herbivorous fish, *Carassius auratus* is an omnivorous fish, and *Hypophthalmichthys molitrix* and *Aristichthys nobilis* are filter-feeding fish. As comprehensively investigated in the present study, it can be observed that half of the OTUs in the water sample can be detected in fish intestines. The ratio of the number of OTUs shared by *Aristichthys nobilis*, *Ctenopharyngodon idellus* and *Carassius auratus* with the water samples accounted for more than 30% of the total OTU samples in water samples, while the ratio of the shared OTU of the *Hypophthalmichthys molitrix* and water samples was only 22%, and the unique OTU in the LC group was relatively the highest in the fish intestinal group. Futher more, it can be seen from the analysis in NMDS analysis, the distance between *Hypophthalmichthys molitrix* group and water group is relatively farthest. Compared to *Aristichthys nobilis*, *Ctenopharyngodon idellus* and *Carassius auratus*, *Hypophthalmichthys molitrix* exhibited some differences in microbiota structure and bacterial species. At the same time, the top 10 genera of abundance in fish intestines could also be detected in the water samples, indicating that there is a strong correlation between fish intestinal microbiota and the water environment. Among these, the fish with a weak correlation was *Hypophthalmichthys molitrix*. From the OTU numbers of each group and the Chao1 index and Shannon index, it could be observed that *Ctenopharyngodon idellus* had the highest microbiota richness and diversity, while the water sample had the lowest richness, indicating that the intestinal microbiota diversity of herbivorous fish is significantly higher than that of omnivorous and filter-feed Fish. These are consistent with the findings reported by some scholars (Larsen et al. 2014; Li et al. 2015; Miyake et al. 2015). From the analysis of the taxonomic composition of the phylum, genus and taxonomic composition differences among groups, it can be observed that fish species have different prevalent microbiota. Firmicutes, *Methylocaldum* and *Bacillus* are the prevalent taxonomic units in the YC and JC groups. Furthermore, *Anaerospora* was the prevalent genus in the LC group, and Proteobacteria and Cyanobacteria had higher relative abundance ratios in the CC group. Moreover, the prevalent taxonomic unit in the water sample group was *Phenylobacterium*, Bacteroidetes and Actinobacteria.

In the classification of vertebrates, fish occupies the most important taxonomic status, and has rich ecological diversity (Parma et al. 2016; Ring et al. 2016). According to the results of the present study, there is a large difference in the structure of the intestinal microbiota among fish species. Compared to mammals, the main microbiota of fish gut are more complicated. The effect of the ambient water environment in direct contact with fish on the intestinal microbiota of fish is significantly greater than that in terrestrial animals and human beings. Baits, drugs and additive are also fed directly into the water to affect the growth of the fish. Some intestinal microorganisms can be used as indicator organisms of the environment. Microorganism indicator species play an important role in detecting changes in the environment of aquaculture water. Most studies have shown that the main microbiota at the phylum level in freshwater fish were Proteobacteria, Firmicutes and Actinobacteria (Wang et al. 2018; Talwar et al. 2018; Nayak 2010). The present study revealed that *Proteobacteria*, *Cyanobacteria* and *Firmicutes* were the main microbiota in the fish intestines and water samples. At the same time, most reports have indicated that the main microbiota at the genus level in freshwater fish were *Aeromonas*, *Pseudomonas* and *Bacillus* (Wu et al. 2012; Meng et al. 2018; Li et al. 2013; Liu et al. 2014). However, the present study revealed that *Synechococcus*, *Anaerospora* and *Bacillus* were the main microbiota in freshwater fish and the water samples. The possible reason is that different water environments can cause differences in fish intestinal microbiota. At the same time, it was speculated that Cyanobacteria, *Synechococcus* and *Anaerospora* are the indicator microbiota of water samples. It is noteworthy that among the top 20 genera with relative abundance, *Cetobacterium* was the unique genus in fish intestines, and *Vogesella* was the unique genus in the water sample. This phenomenon indicates that *Cetobacterium* is likely to be in fish intestines, and that *Vogesella* is not suitable for survival in fish intestines.

### Accession number

SRP269121.
